# Supplementary figures and images for: Depolymerization of SUMO chains induces slender to stumpy differentiation in T. brucei bloodstream parasites
Source: PLoS Pathog. 2024 Apr 18;20(4):e1012166. doi: 10.1371/journal.ppat.1012166 (PMC11060531; doi:10.1371/journal.ppat.1012166)

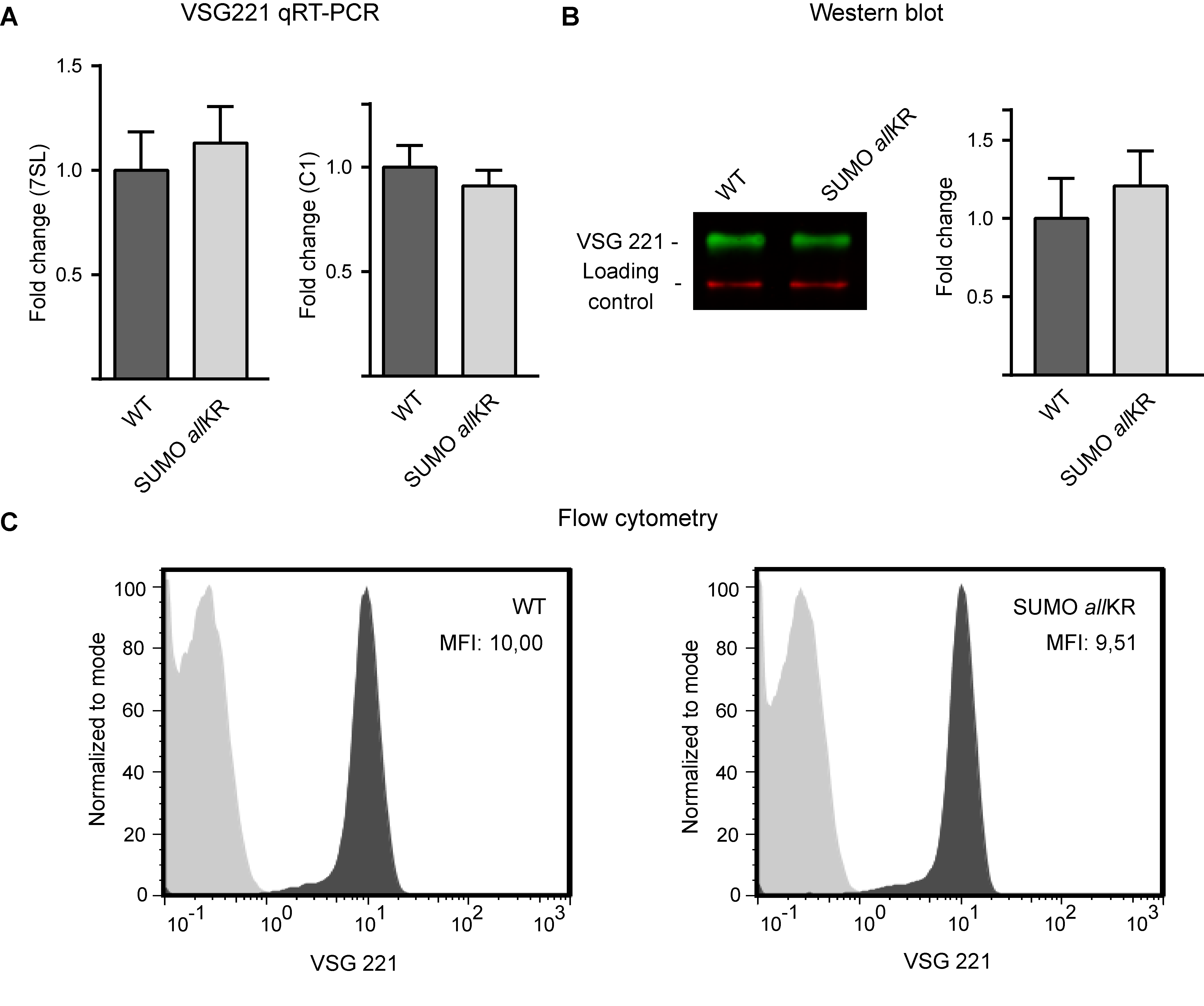

Supplement: S1 Fig — (A) Quantification of RNA transcript levels corresponding to the VSG221 gene expressed in WT and SUMO allKR parasites. Transcript levels were determined using qRT-PCR and normalized against 7SL and C1 (n = 3). (B) VSG221 expression levels were evaluated by Western blot analysis in total cell extracts with anti-VSG221 antibodies and tubulin as a loading control. Experiments were performed at least in triplicates and one representative image is shown. Quantification of band intensities was performed using ImageJ. (C) The density of the VSG coat in WT and SUMO allKR parasites was analyzed in fixed cells with anti-VSG221 antibodies and flow cytometry. Approximately 50000 events were captured. Representative flow cytometry histograms normalized to mode are shown. MFI: median fluorescence intensity. (TIF) [file ppat.1012166.s001.tif]

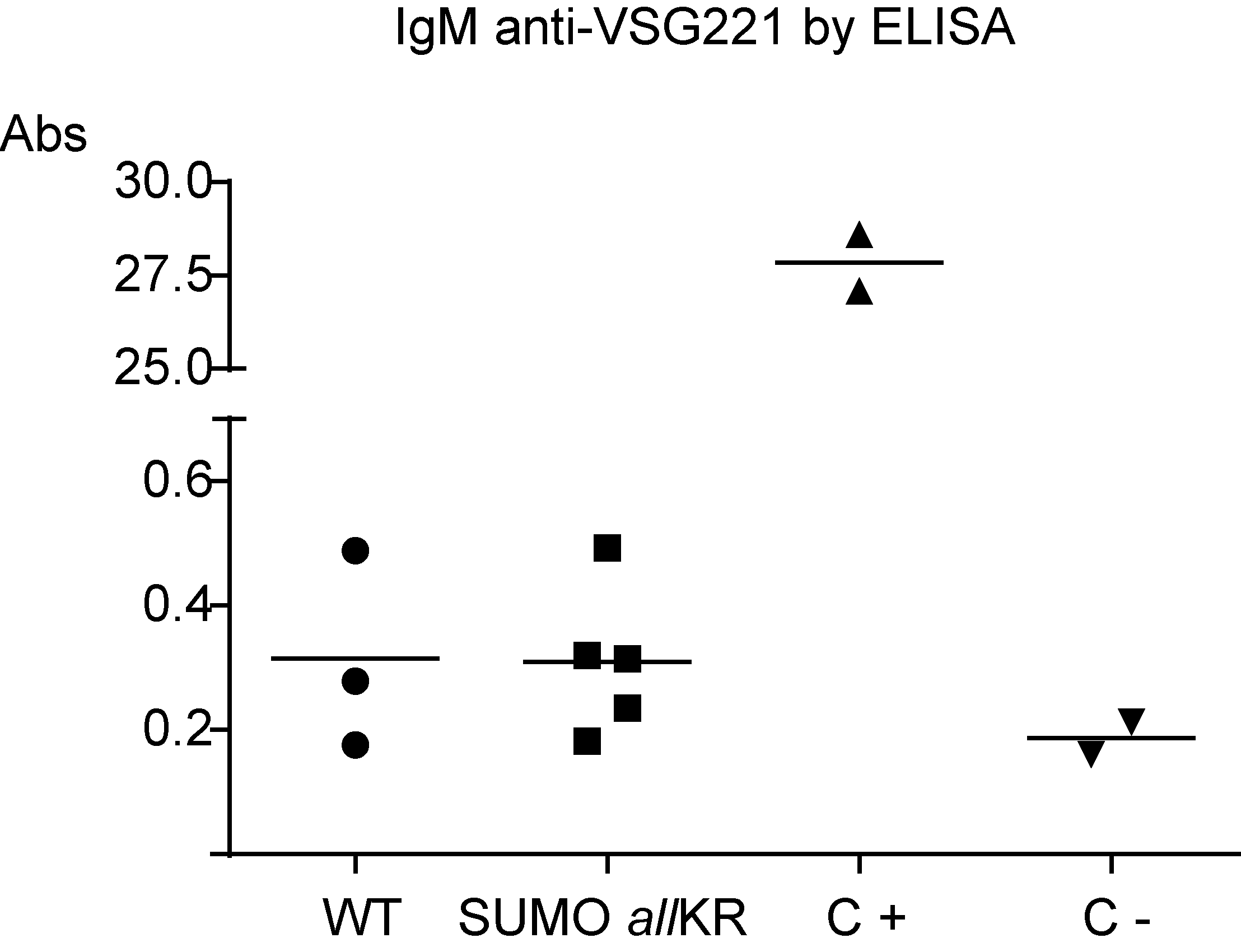

Supplement: S3 Fig — Detection of IgM antibodies against VSG221 in the serum of mice infected with WT or SUMO chain mutant (SUMO allKR) parasites at the first peak of parasitemia by ELISA. Serum samples were diluted 1:5, and absorbance values (Abs) were measured at 450 nm. C+: positive control; C-: negative control. (TIF) [file ppat.1012166.s003.tif]

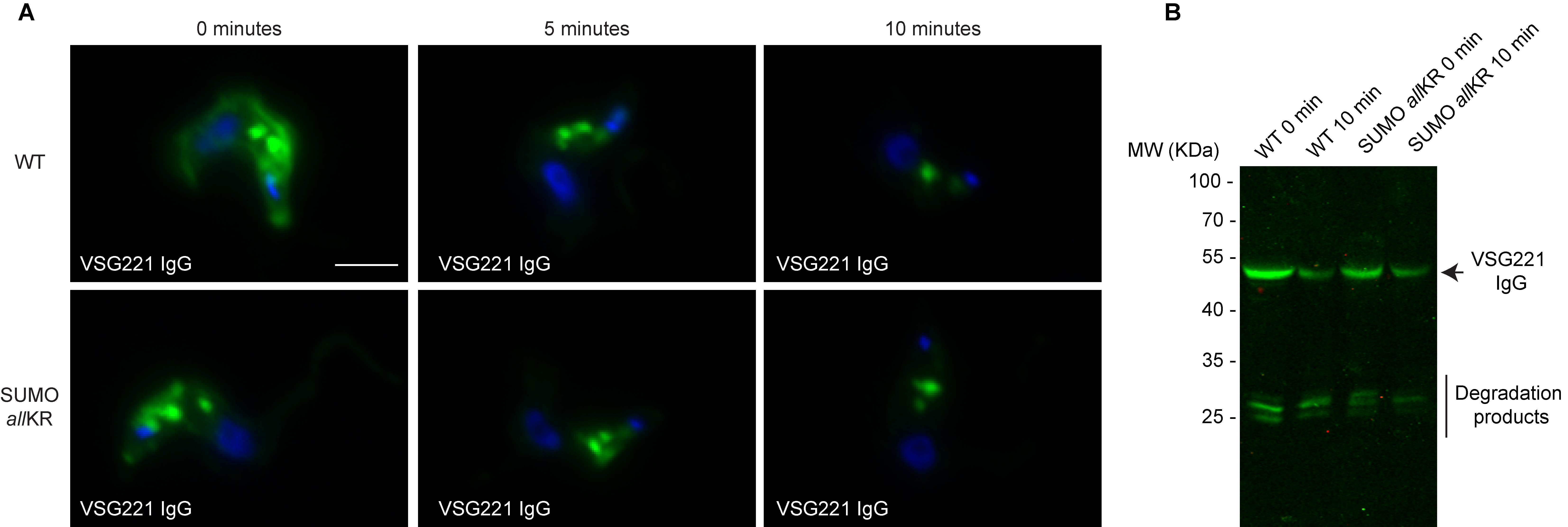

Supplement: S4 Fig — WT or SUMO chain mutant (SUMO allKR) parasites were first incubated with anti-VSG221 IgG at 4°C in HMI-9, followed by an incubation at 37°C for 5 and 10 minutes. (A) Parasites were fixed and stained with anti-mouse-Alexa Fluor 488 (green) and DAPI (blue). (B) Whole-cell extracts (corresponding to 1x10^7^ cells) were obtained after the 10-minute incubation at 37°C and boiled in Laemmli’s sample buffer. Proteins were separated by SDS-PAGE and transferred to a nitrocellulose membrane, followed by immunoblotting with anti-mouse-Alexa Fluor 790. (TIF) [file ppat.1012166.s004.tif]

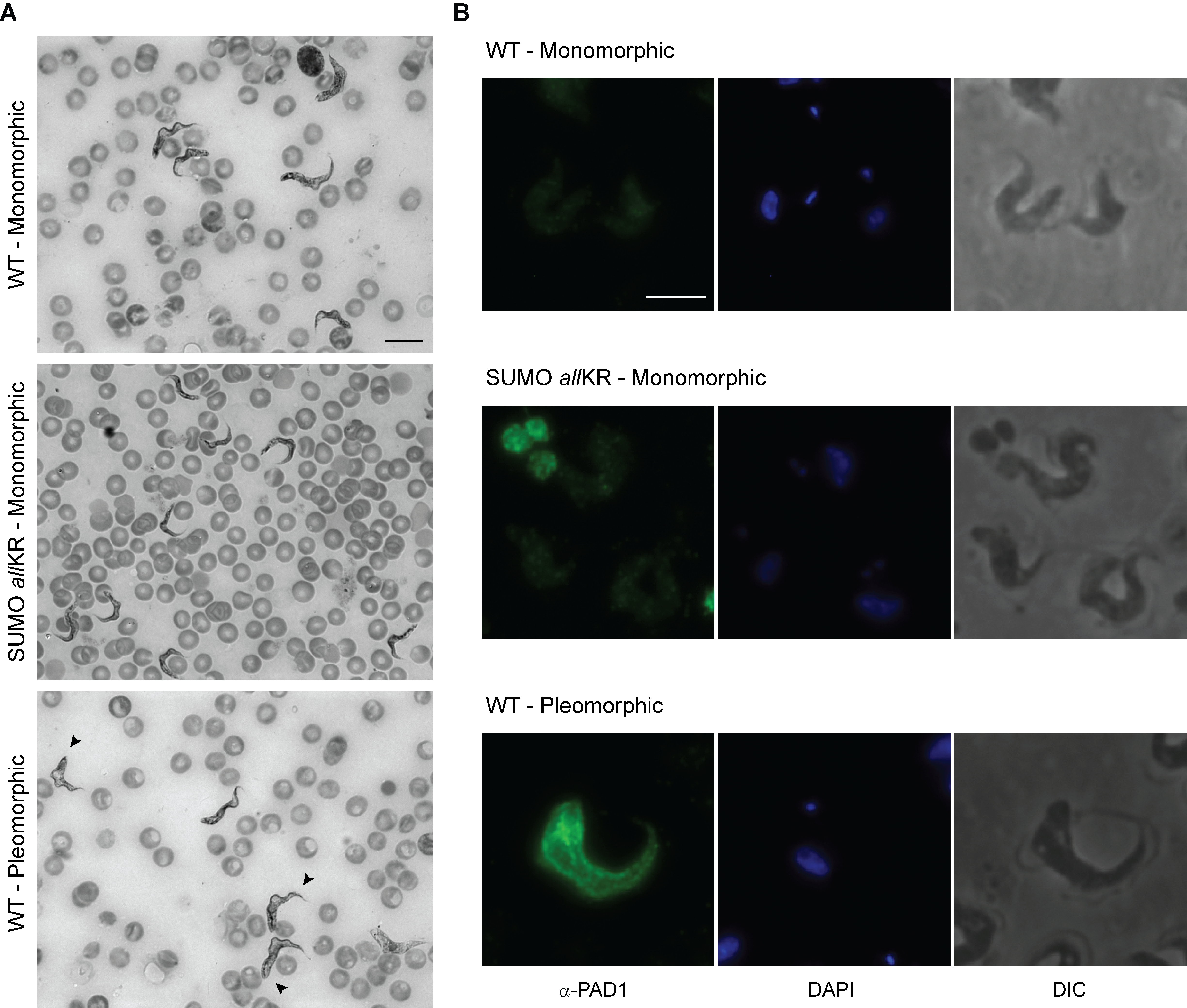

Supplement: S5 Fig — Representative images of methanol-fixed blood smears of infected mice on the peak of parasitemia (at 5 dpi) stained with (A) Giemsa; (B) anti PAD1 antibodies (green) and DAPI (blue). Stumpy cells from pleomorphic parasites are shown as positive control (arrowhead). Scale bar 10 μm. (TIF) [file ppat.1012166.s005.tif]

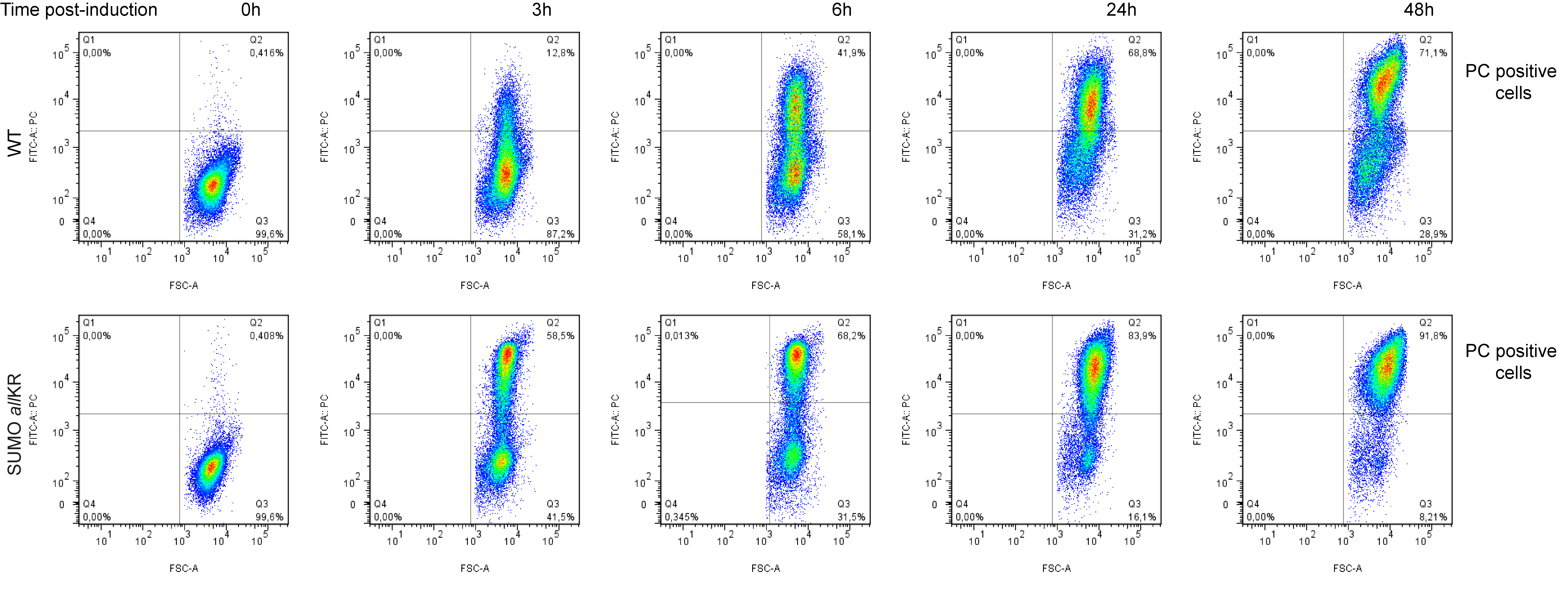

Supplement: S6 Fig — WT or SUMO allKR parasites were harvested from semisolid HMI-9 agarose plates and incubated with 6 mM cis-aconitate in SDM-79 at 28°C. Parasites were collected by centrifugation after different time points, fixed and stained with anti-EP FITC. Samples were analyzed by flow cytometry. (TIF) [file ppat.1012166.s006.tif]

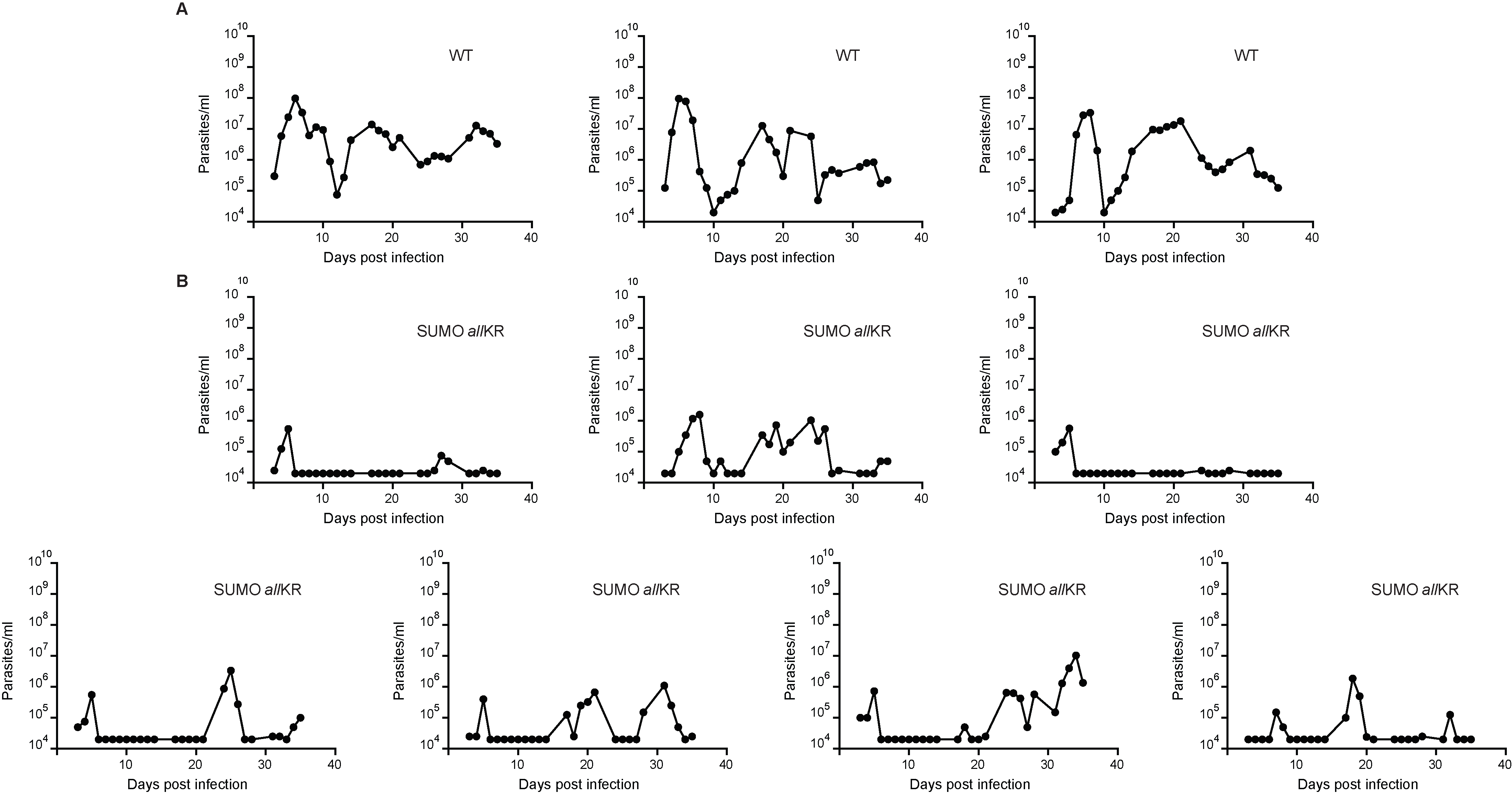

Supplement: S7 Fig — Time course parasitemia in mice infected with (A) WT or (B) SUMO allKR parasites. (TIF) [file ppat.1012166.s007.tif]
