# Supplementary material for: Depolymerization of SUMO chains induces slender to stumpy differentiation in T. brucei bloodstream parasites
Source: PLoS Pathog. 2024 Apr 18;20(4):e1012166. doi: 10.1371/journal.ppat.1012166 (PMC11060531; doi:10.1371/journal.ppat.1012166)
Supplement: Attachment — Submitted filename: Rebuttal letter.pdf [file ppat.1012166.s010.pdf]

Instituto de Investigaciones
Biotecnológicas
IIB_UNSAM

San Martin, March 15th 2024

Michael Boshart
Academic Editor
PLOS Pathogens

Dear Dr Boshart,

We appreciate you taking the time to read through and comment on our manuscript entitled “Depolymerization of SUMO chains induces slender to stumpy differentiation in *T. brucei* bloodstream parasites” (PPATHOGENS-D-24-00124). We have carefully reviewed your suggestions and made the appropriate changes accordingly.

As requested by the reviewers, we have fully documented all experiments, including the statistical evidence, and improved the presentation of the data and figures. You can follow all changes in our point-by-point answer.

We have also revised the conclusions and discussion to specifically emphasize that:

1) Differentiation into stumpy forms is not exclusively regulated by cell density-dependent mechanisms and various stimuli and stressors can trigger the formation of stumpy cells. Furthermore, it has been hypothesized that *T. brucei* evolved mechanisms that merged stress responses with pathways controlling differentiation (Quintana et al., 2021).
2) Numerous studies have shown that SUMO is essential for the maintenance of cell homeostasis when the cell is exposed to endogenous or environmental stress (Vertegaal et al., 2022).
3) Based on our results, since SUMO chain mutant pleomorphic parasites are primed for differentiation from bloodstream slender to stumpy forms we discuss the possibility that the absence of SUMO chains might reduce the threshold required for the stress activated differentiation program.

Below we provide the point-by-point responses. All modifications in the manuscript can be tracked.

Reviewer #1:

F1: Panel B - comment on residual possible SUMO adducts. Do the authors consider that these represent monoSUMOylated adducts?

Thank you for pointing this out. The reviewer is correct, the bands observed in the Western blot can be attributed to the ability of SUMOallKR to be conjugated as a monomer to target proteins at one or more lysine residues, as shown schematically in the original Fig1A. To further clarify this point, we have slightly modified the text (page 6, lanes 131-134) to include the following:

"SUMOallKR can be processed and conjugated as a monomer to target proteins at single or multiple lysine residues, resulting in mono- or multiSUMOylated adducts (TbSUMOallKR, Figure 1A) that show a reduced intensity of the SUMOylation pattern in Western blot (Figure 1B),…"

In **panel D** the authors should comment on the punctate staining and that the vast majority of the signal is likely to derive from unconjugated SUMO. I'm also not 100% convinced that the nuclear focus at panel D bottom might not be at the cytoplasmic face of the nucleus?

As suggested by the reviewer, we have added a sentence to the manuscript (page 6, lanes 138-141) that refers to the fact that in parasites unable to form SUMO chains, not only is there a decrease in the number of parasites with a clear nuclear focus, but also a punctate signal is observed in the cytoplasm, most likely due to the unconjugated fraction of SUMO.

F2: Make clear if the graphs in panel B are representative examples or collated data?

In the original version of the manuscript, the graphs in Figure 2, panel B, corresponded to representative examples of the parasitemia profiles observed for wild-type and SUMO chain mutant parasites, while Supplementary Figure 2 showed the individual parasitemia values for all mice infections. Taking into account the recommendations of the 3 reviewers, we have now modified Figure 2, panel B, to show the differences between mice in the main figure (new Figure 2, panel B).

F3: Panel A - WT seems to have very low G0 proportion; I tend to think of this as being closer to 80% in well growing cultures.

The proportion of 1K 1N parasites in culture is close to 70% (Forsythe et al., Mol Biochem Parasitol. 2009;164(2):131-136), whereas this number tends to be

lower in vivo (Trindade et al., Nat Commun. 2022;13(1):7548). These differences are probably due to the high multiplication capacity of parasites in the blood which is accompanied with a higher proportion of parasites with 2K 2N configuration.

I FOUND the ANNOTATION OF **PANEL C** SOMEWHAT UNCLEAR. FOR EXAMPLE THE TOP category was near invisible on my screen.

As suggested by the reviewer, we have changed the color palette for a better visualization of the data.

and I have no idea what Grumpy is. Overall I felt the data for this panel deserve some better discussion.

In the original version of the manuscript we mentioned that "Among the upregulated transcripts, we found 6 noncoding RNA in the top 10 and Tb927.10.12080, a reported target of grumpy whose overexpression was associated with stumpy development [7]." We indicate now that grumpy is a long non coding RNA (page 7, lane 171) and the reader can find additional information in the Discussion section of the original manuscript (page 12, lanes 279-285): "On the other hand, it was noticed that a number of noncoding RNAs are found among the most abundant transcripts. It was recently described that a lncRNA, which gives rise to a small nucleolar RNA called grumpy, binds to a number of mRNAs involved in the transition to stumpy forms and promotes their expression [7]. Interestingly, one of the mRNA targets of grumpy, whose overexpression has been experimentally confirmed to trigger prematur differentiation, is also upregulated in our chain mutants, supporting the notion that these parasites are committed to the differentiation pathway."

I note that the data in **Table S1** use old 427 annotations and it would be helpful if these were updated or included the 927 ortholog accession.

We agree with the reviewer and so a new Table S1 has been submitted to update 427 annotation and to include the 927 ortholog accession.

Also, morphologically these do not look to be stumpy cells and is worth comment?

It is in fact very interesting that the loss of polySUMOylation increases the sensitivity of monomorphs to the CA signal without generating full stumpy forms. We have now included an additional Supplementary Figure (Fig S5) to show this data as requested by reviewers #2 and #3.

FS3: I'm not fully confident that the analysis here truly represents a test for the entire immune system. I think the point is well taken that the attenuation is not due to some massive immune response but could be diluted a little.
We agree with this comment and have changed the text (page 7, lane 158 and page 11, lane 269) and the legend of the Fig S3 (page 31, lane 755) replacing the sentence stating that we evaluated "host humoral immune response" with a sentence stating that we evaluated "anti-VSG IgM antibody response".

Reviewer #2:

The data in **figure 2b and S2** are unclear in the current format. The text states "In contrast, mice infected with SUMO chain mutants (n=17) showed two or three waves of parasitemia and in some cases even cleared the infection"
Based on the comments of all reviewers we have modified Fig2 panel B and Fig S2 to present the results more clearly.

• How many mice cleared the infection? It is just one? The one shown in group 3? *The reviewer is correct. Only one mouse cleared the infection and this is now mentioned in the text (page 7, lanes 153-155) as well as in the legend to the Fig S2 (page 31, lane 753).*

• How many mice died as a result of the infection with the mutant line? Please indicate on the plots. *Two mice died during this experiment (at day 10 p.i.) and this is now indicated on the plots with an asterisk and Is mentioned in the legend to the Fig S2 (page 31, lanes 752-753).*

- Was the experiment ended on day 11? Or did the mice die due to the infection at this point? Or did they clear the parasites at this point? *The experiment ended on day 11 indeed. During this time parasitemia was monitored daily. This has been clarified in the text (page 7, lanes 150-151).*
- It would be clearer to show the variation between mice in the main figure. Please add the data points/error bars to the trends in figure 2b. *Taking into account the recommendations of the 3 reviewers, we have now modified Figure 2, panel B, to show the differences between mice in the main figure (new Figure 2, panel B). Mice were grouped according to the different parasitemia profile. Results are shown as the means and their corresponding SEM.*

  In reference to monomorph infection, authors state "At the peak of parasitemia, we observed cells that could not be recognized as genuine

stumpy parasites based on their morphology or biochemical markers (not shown) [19].” Please show these data, state the biochemical markers (PAD1?) and make clear the timepoint and mice these sample were taken from. It is necessary to show these data as it impacts the interesting finding that the loss of polySUMOylation increase the sensitivity of monomorphs to the CA signal without generating full stumpy forms. *Thank you for this suggestion. We have now included an additional Supplementary Figure 5 (Fig S5) to show the morphology of the cells with Giemsa staining and the detection of the cell surface stumpy marker PAD1 by IF.*

Other points:

• In **figure 1. Panel B and C** are labelled incorrectly. *Thank you for pointing this out. The legend to Figure 1 has been corrected.*

• What is meant by “three-dimensional immunofluorescence” in reference to fig **1D**? It’s not clear in the methods what 3D is referring too. *Thank you for pointing this out. With “3D immunofluorescence” we refer to the fact that images were acquired as 3D z-stacks, deconvoluted and projected into 2D using a maximum intensity projection, as previously done in López Farfán et al., 2014. We have included a description of this procedure in the methods section (page 17, lanes 413-415) and deleted 3D to avoid confusion.*

• In **figure 3a**, the plot shows “*” and legend says “**, -<0.01”, please clarify. *Thank you for pointing this out. The legend to Figure 3A has been corrected (page 29, lanes 694-695).*

• In **figure 3B**, does M1-M5 indicate mouse 1- mouse 5? *Yes. We are sorry we omitted this abbreviation which has now been added in the legend to the figure (page 28, lane 680).*

• Please state the time points for the data in **figure 3b and c**. when was RNA collected? *RNA was collected on day 5 p.i. This has been indicated in the legend to the Figure 3 (page 28, lane 681 and page 28, lane 684).*

• For **figure 3D and E**, please indicate replicates, exact counts and error bars. *Replicates, exact counts and error bars have been added in the legend to the Figure 3 as requested (page 28, lanes 690 and page 29 lanes 693-695).*

• In **figure 5A** please add error bars. *Following the recommendations of the reviewers, we have modified Figure 5 similar to what has been done for*

Figure 2. Parasitemia values are shown as the means with their corresponding SEM. Differences between mice infected with WT and SUMO allKR parasites are shown in the same graph.

- For **figure 5C, D and E**, please indicate the time point the sample was made from, when in the peak? Were these taken on the same day for WT and mutants? *Samples were collected at the first peak of parasitemia (6 dpi) and this is now mentioned in the legend to the figure (page 30, lanes 721-722).*

  • In **figure 5E**, is this just one replicate? replicates should be shown. *The experiment was performed in triplicate. The modified panel E of Figure 5 shows the mean and SEM of the experiments and legend to the figure was modified accordingly (page 30, lane 722).*

  • How were "intermediate cells" defined in **5E**. *Intermediate cells are defined by weak labelling with anti-PAD1 observed mainly in vesicles. This has been illustrated with representative images at the bottom of the graph, and it has also been clarified in the legend to the figure (page 30, lanes 720-721).*

  • The **discussion** states "In monomorphic parasites, mice infected with SUMO chain mutants showed relapsing and remitting waves of parasitemia, reaching densities not higher than 108/ml and clearing the day after." Plots in 2B and supplementary show parasitaemia reaching 1x10^9/ml. It isn't clear which mice cleared the parasites currently. *We agree with the reviewers observation. In fact parasitemia reaches 1x10^8-1x10^9/ml and the text was modified accordingly (page 11, lanes 257). Regarding the indication of which mouse cleared the infection, this has been now mentioned in the main text of the manuscript (page 7, lanes 153-155) and in the legend to the Figure S2 (page 31, lane 753).*
- Please deposit RNA-seq data to online repository and reference the accession number. *"The RNA-seq data generated and analyzed during the current study are available in the GEO repository under accession number GSE261736 (https://www.ncbi.nlm.nih.gov/geo/query/acc.cgi?acc=GSE261736)". Reviewer can access the data using the confidential private token ytingymqnzipnmh.*

<u>Reviewer #3:</u>

Introduction

• Do Trypanosoma brucei spp parasites only live in the blood of infected animals? *Thank you for pointing this out. We have modified the text to mention that T. brucei can colonize multiple host tissues, including the blood, lymphatic system, skin, adipose tissue, and eventually the central nervous system (page 3, lanes 59-61).*

Results

• 'However, this difference does not affect VSG mRNA or protein levels (**Figure S1**), suggesting that mono- and/or multi-SUMOylation are sufficient to promote VSG expression.' Do you mean that the absence of poly-SUMOylation does not affect VSG mRNA or protein levels? *The reviewer is correct. We have eliminated the second part of the sentence. (page 6, lanes 141-142)*

• 'At the peak of parasitemia, we observed cells that could not be recognized as genuine stumpy parasites based on their morphology or biochemical markers (not shown) [19]' This is important and I think the reader would appreciate to see these cells. *Thank you for this suggestion. As requested also by reviewer#2, we have included an additional Supplementary Figure (Fig S5) to show the morphology of the cells stained with Giemsa and the detection of the cell surface stumpy marker PAD1 by IF.*

• 'In addition, PAD1 and 12 transcripts that are highly expressed in stumpy forms were also upregulated in these cells [20, 21].' Were transcripts of the FHR and/or enzymes of the Ox-Phos pathway also upregulated in these cells? *No, we have not observed differential expression of FHR or enzymes of the Ox-Phos pathway.*

• 'Overall, these results suggest that the absence of polySUMOylation may stimulate the formation of stumpy-like cells, rendering monomorphic bloodstream parasites more sensitive to CA-triggered differentiation and that the underlying mechanism is upstream of AMP signalling.' Please rephrase. These data do not show any stimulatory effects in absence of polySUMOylation. *We have rephrased this sentence to state that the absence of polySUMOylation renders monomorphic parasites more sensitive to CA-triggered differentiation. (page 8, lanes 194-196).*

• A bona fide comparison between different types of induction of ST differentiation would have more clearly assessed the position of the

polySUMOylation effect along the differentiation pathway. For instance, a comparison between cAMP and spent medium or basement membrane extract medium. We have shown that chain mutant pleomorphic parasites are primed for differentiation to stumpy in vitro and in vivo during mice infections. *We agree with the reviewer that it would be interesting to analyze how polySUMOylation interacts with the different components of the quorum sensing differentiation pathway (such as GPR89 (Rojas et al., 2019), the proteases oligopeptidase B and metallocarboxypeptidase 1 (Tettey et al., 2022), TOR4 (Saldivia et al., 2013), AMPK (Saldivia et al., 2016) and others (Mony et al., 2014). However, this aspect is currently beyond the scope of this manuscript.*

• How could these monomorphic mutants differentiate into PCF upon CA induction without PAD1 and PAD2 detected at their surface? *Monomorphic, slender trypanosomes that do not express detectable levels of PAD proteins can differentiate in response to CA, although this process is not synchronous and takes 36-48 hours to complete. It has been speculated that signaling is probably controlled by transporters other than PAD1 or PAD2 due to the high concentrations of CA required for differentiation (Czichos J et al., 1986 y Dean S et al., 2009)*

• 'As shown in **Figure 4D and 4E**, chain mutant parasites had a significantly higher proportion of cells expressing PAD1 (74% ± 2%), while only 35% ± 6% of WT cells showed expression of PAD1 at the same time point.' It is not easy for me to detect any single PAD1+ cell in the picture presented in Fig4D for WT cells. *We thank the reviewer for this reference. In the original version of this manuscript, we were less stringent in categorizing a cell as PAD+, as we scored a cell when IF signal was present either on the membrane or in vesicles. As noted by the reviewer, and in contrast to the SUMO chain mutants, WT cells show no PAD1 labelling on the surface and only weak signal in vesicles. We now present a requantification of the same experiment in which a cell is scored as PAD+ only if it shows labelling on the surface.*

• Could the lower parasitemia observed during infections with the SUMO allKR strain in **Fig 5B** result from an increased level of parasite extravasation? *While it is true that an increase in parasite extravasation would lead to a decrease in parasitemia, we did not investigate this possibility because all results taken together suggest a role for chains in differentiation.*

‘Our results thus clearly show that the absence of SUMO chains is a signal for stumpy development during infections.’ This claim is not correct. The data presented here do not show that the absence of SUMO chains is a signal for stumpy development. Please, modify this sentence. *We agree with the reviewer that our claim is based on speculations. We have deleted "signal" and state that "the absence of SUMO chains facilitates stumpy development during infections." (page 10, lanes 224-225).*

Discussion

• ‘Our conclusion is based on several experimental findings: 1) the outcome of the infection with SUMO chain mutants BSF; 2) the expression of stumpy markers; and 3) the accelerated rate of differentiation to PF following cis-aconitate exposure.’ Please dissociate this result overview according to the pleo Vs monomorphic strains, because each strain displayed different phenotypes. *Following this suggestion we have dissociated the overview for monomorphic and pleomorphic parasites (page 10, lanes 230-235): "Our conclusion is based on several experimental findings: 1) attenuated virulence for monomorphic and pleomorphic parasites; 2) stumpy-like transcriptome profile in monomorphic parasites and premature PAD1 protein expression in pleomorphic parasites; and 3) the accelerated rate of differentiation to PF following cis-aconitate exposure in monomorphic and pleomorphic parasites."*

• ‘abrogation of SUMO chains has reverted the typical behaviour of monomorphic strains in vivo, acquiring characteristics of pleomorphic parasites,’ Here again, you may tone down your statement. *Following this suggestion we have replaced this statement with the following (page 11, lanes 262-264): "Thus, it appears that the abrogation of SUMO chains has attenuated the virulence of monomorphic strains in vivo, prolonging mouse survival and decreasing parasitemia."*

• ‘Remarkably, these intermediate forms were only observed in vivo, while in vitro cultured SUMO allKR parasites were negative in all assays used to evaluate stumpy characteristics (morphology, mild acid resistance, mitochondrial activity, cell cycle status and expression of stumpy markers; data not shown) [19] except for differentiation using CA stimulus.’ If the only similarities with stumpy forms are observed at the transcriptomic level and only

partially, could you even refer to these cells as ‘intermediate phenotypes’? These results are nice and it is not necessary to over-interpret the phenotype. *We understand the reviewer's comment and have removed the phrase "intermediate form”. However, we want to emphasize that the recent characterization of the differentiation process from slender to stumpy using scRNAseq (Larcombe et al., 2023) has shown that transcriptional changes precede morphological changes. In fact, parasites with slender morphology can be identified as already arrested in the cell cycle and with a transcriptional profile indicating their commitment to differentiate into stumpy forms.*

Figures

• A graphical abstract or a schematic model summarizing your findings would be a plus. *We have designed a schematic model summarizing our findings (Figure 6)*

• Please, add SD bars in plots in Fig 2, 3 and 5. *SD were added to Fig 2, 3 and 5.*

• Please, add scale bars in picture panels in Fig 1, 3, 4 and 5. *Scale bars were added to picture panels in Fig 1, 3, 4 and 5.*

• Please, clearly mention the total numbers of cells, mice and replicates in all legends. *Total number of cells, mice and replicates were mentioned in all legends.*

Again, I would like to thank you and the Reviewers for helping us improve the quality of our work.

Sincerely,

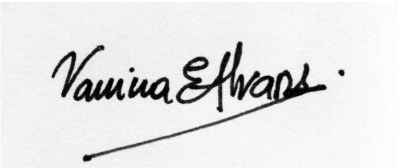

Vanina Alvarez, PhD
Professor
Argentinian National Research Council. CONICET.
Instituto de Investigaciones Biotecnológicas.
Universidad Nacional de San Martín
